# Prevalence and genetic diversity of tick-borne encephalitis virus in ixodid ticks from specific regions of northwestern Russia

**DOI:** 10.1371/journal.pone.0314385

**Published:** 2025-01-30

**Authors:** Alena A. Sharova, Nikolay K. Tokarevich, Regina R. Baimova, Olga A. Freylikhman, Islam A. Karmokov, Ekaterina G. Riabiko, Gelena A. Lunina, Roman V. Buzinov, Olga V. Sokolova, Lidia V. Buts, Lubov A. Bespyatova, Liliya A. Bubnova, Olga S. Safonova, Elena L. Kalinina, Andrey I. Stankevich, Rose Vikse, Ashild K. Andreassen, Anna S. Gladkikh, Majid Forghani, Anastasia S. Gritseva, Margarita R. Popova, Edward S. Ramsay, Vladimir G. Dedkov

**Affiliations:** 1 Saint Petersburg Pasteur Institute, St. Petersburg, Russia; 2 North-Western State Medical University named after I. I. Mechnikov, St. Petersburg, Russia; 3 North-West Public Health Research Center, St. Petersburg, Russia; 4 Federal Service for the Oversight of Consumer Protection and Welfare Arkhangelsk Oblast, Arkhangelsk, Russia; 5 Federal Service for the Oversight of Consumer Protection and Welfare Leningrad Oblast, St. Petersburg, Russia; 6 Institute of Biology, Karelian Research Centre, Russian Academy of Sciences, Petrozavodsk, Russia; 7 Center for Hygiene and Epidemiology, Republic of Karelia, Petrozavodsk, Russia; 8 Federal Service for the Oversight of Consumer Protection and Welfare Pskov Oblast, Pskov, Russia; 9 Center for Hygiene and Epidemiology, Pskov Oblast, Pskov, Russia; 10 Norwegian Institute of Public Health, Oslo, Norway; 11 Institute of Natural Sciences and Mathematics, Ural Federal University, Ekaterinburg, Russia; 12 Martsinovsky Institute of Medical Parasitology, Tropical and Vector Borne Diseases, Sechenov First Moscow State Medical University, Moscow, Russia; University of Padua, ITALY

## Abstract

Russia is a country with a high incidence of tick-borne encephalitis (TBE). In northwestern regions of Russia, 110 TBE cases were registered in 2021. The largest numbers of TBE cases were registered in the Arkhangelsk region and St. Petersburg. TBEV seropositivity among healthy individuals, including the unvaccinated population in northwestern Russia, was found in 12.2% of studied samples, indicating active TBEV circulation. The prevalence of TBEV is 2.4% in the two tick species most common in northwestern regions of Russia, *Ixodes ricinus* and *Ixodes persulcatus*. However, there is still no comprehensive data on the molecular characterization and phylogenetic analysis of the circulating TBEV strains. The purpose of the study was to determine the prevalence of TBEV and to identify its subtypes in ixodid ticks collected in specific areas of northwestern Russian regions. Phylogenetic analysis of E protein sequences of ten obtained strains showed that they all belong to the Siberian subtype, which were clustered into two groups: the most numerous Baltic group, clusteron 3D; and the Vasilchenko group. However, some unique isolates may form new clusterons.

## 1. Introduction

Tick-borne encephalitis virus (*TBEV*, *Orthoflavivirus encephalitidis*) belongs to the *Flaviviridae* family, *Orthoflavivirus* genus [1]. TBEV is a positive-sense enveloped virus with a single-stranded RNA (+ssRNA) and genomic length of about 11 kb [2]. The TBEV genome represents a 5′-cap plus a single open reading frame (ORF) flanked by 5′ and 3′ untranslated regions. The ORF codes for a single polyprotein that is co- and post-translationally cleaved by viral and cellular proteases into three structural (C, prM, E) and seven non-structural proteins (NS1, NS2A, NS2B, NS3, NS4A, NS4B, NS5) [3]. Among the three structural proteins, a fragment of glycoprotein E serves as a phylogenetic representative which is usually employed for phylogenetic analysis [4]. Based on it, TBEV species may be divided into seven subtypes: European (TBEV-Eur); Siberian (TBEV-Sib); Far-Eastern (TBEV-FE); Obsky (TBEV-2871 (TBEV-Ob)); Himalayan (TBEV-Him); Baikalian-1 (TBEV-178-79 (TBEV-Bkl-1)); and Baikalian-2 (TBEV-886-84 (TBEV-Bkl-2)) [5].

The main vectors of TBEV are *Ixodes ricinus* and *Ixodes persulcatus* ticks. TBEV is an arbovirus, which can infect humans and a wide range of animals [6, 7], causing severe neuroinvasive infection termed tick-borne encephalitis (TBE), which is sometimes fatal. This infection is endemic in Europe and northeastern Asia due to wide distribution of ixodid ticks and is considered to be the most important arboviral disease in these regions [3].

Russia is a country with high TBE incidence. In 2022, 1957 TBE cases (including 60 fatalities) were registered in 48 different regions of the country [8]. In the Northwestern Federal District, 110 TBE cases were registered in 2021, including three deaths (two in the Arkhangelsk region, one in the Vologda region). The largest regional numbers of TBE cases were registered in the Arkhangelsk region and Saint Petersburg (29 and 25 cases, respectively) [9].

TBEV seropositivity among healthy individuals, including the unvaccinated population in northwestern Russia, indicates active TBEV circulation as well as human contact with the virus. Specifically, anti-TBEV antibodies (IgG) were detected in 12.2% of residents of northwestern Russia, including: 14.1% in the Leningrad region; 13.0% in the Republic of Karelia; 12.6% in the Komi Republic; 11.2% in the Pskov region; and 6.8% in the Arkhangelsk region [10].

In 2022, more than 500,000 people in Russia sought medical help after a tick bite [8]. However, a lack of specific data for northwestern Russia (TBEV prevalence, circulating viral subtypes, etc.) is concerning. The aim of this study was to determine TBEV prevalence, and to identify relevant subtypes, in ixodid ticks collected in specific areas of northwestern Russia.

## 2. Materials and methods

### 2.1 Sample collection and RNA extraction

**Fig 1:**
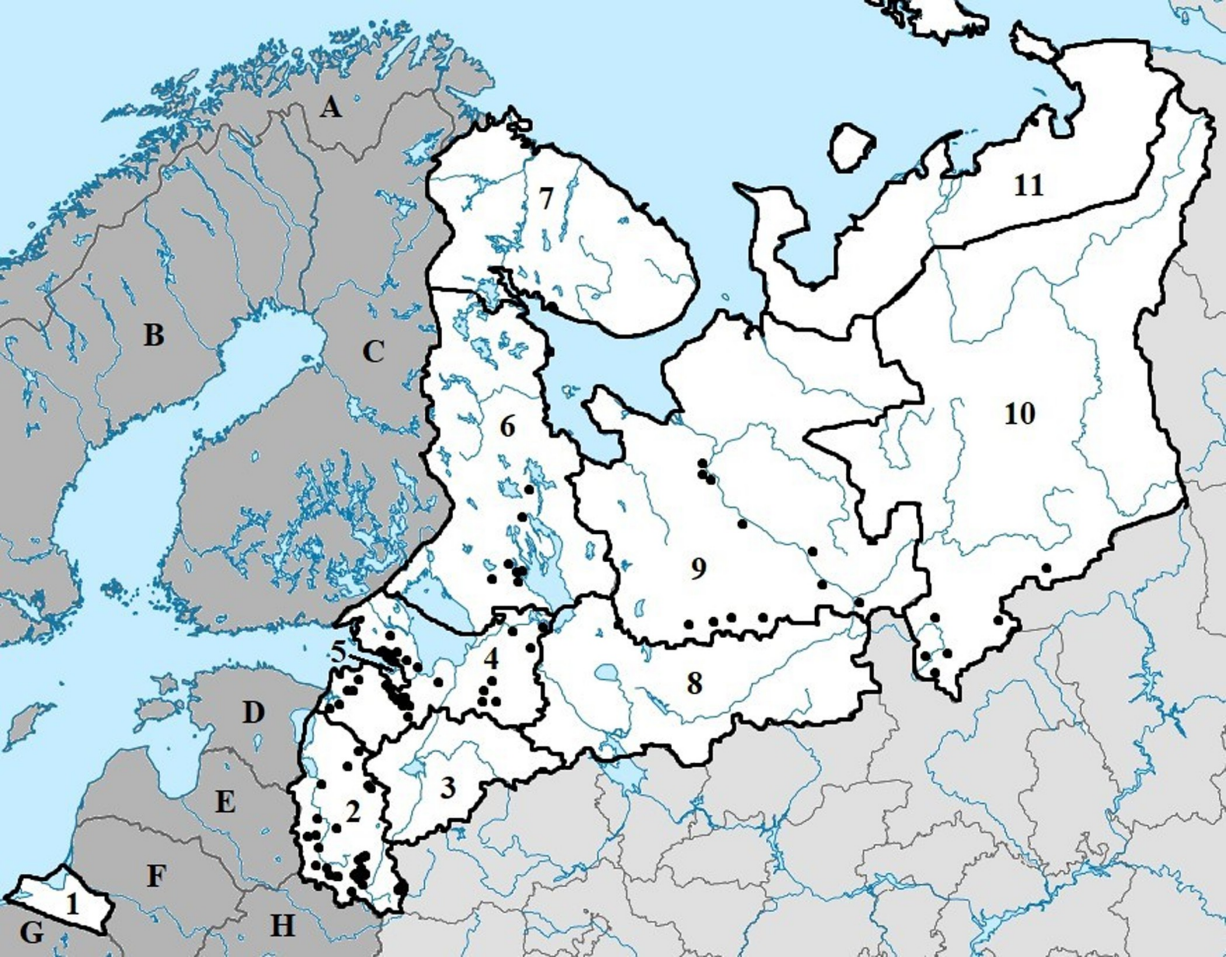
Tick collection points. A—Norway, B—Sweden, C—Finland, D—Estonia, E—Latvia, F—Lithuania, G—Poland, H—Republic of Belarus. Regions: 1—Kaliningrad region, 2—Pskov region, 3—Novgorod region, 4—Leningrad region, 5—St. Petersburg, 6—Republic of Karelia, 7—Murmansk region, 8—Vologda region, 9—Arkhangelsk region, 10—Komi Republic, 11—Nenets Autonomous Area. Reprinted from [https://upload.wikimedia.org/wikipedia/commons/1/1c/Outline_Map_of_Northwestern_Federal_District.svg?uselang=ru] under a CC BY license. The drawing is similar, but not identical, to the original image and is for illustrative purposes only.

We studied 2,812 questing ixodid ticks (*I*. *persulcatus*, *I*. *ricinus*) collected in 93 spots located in 6 northwestern regions of Russia from 2015 to 2022, specifically: 11 spots in the Arkhangelsk region; 27 spots in the Leningrad region; 31 spots in the Pskov region; 8 spots in the Republic of Karelia; 6 spots in the Komi Republic; and 10 spots in Saint Peterburg (Fig 1 and S1 Table). The study was conducted during routine surveillance of TBEV and vector competence in accordance with Rospotrebnadzor regulation No78 "Prevention of infections transmitted by ixodic ticks" (Registered with the Ministry of Justice of the Russian Federation on 12.02.2016, No. 41065). All ticks were collected from vegetation during field visits by flagging (flag size: 0.6 m × 1.0 m), as described in Bugmyrin et al. [11]. The species and sex of ticks were determined based on morphology following earlier recommendations [12]. The ticks were classified (species, sex, developmental stage) and stored alive in a wet chamber until homogenization. Ticks were homogenized individually using a FastPrep-24 homogenizer (MP Biomedicals, USA) for 2 min in 400 μL of phosphate-buffered saline (pH 7.0, Dako, Denmark) before extraction. Following centrifugation at 14,000 g for 2 min, total nucleic acids were extracted and purified from the supernatant by the RIBO-prep DNA/RNA extraction kit (AmpliSens®, Russia) according to manufacturer recommendations. DNA/RNA was eluted with 50 μL of elution buffer (AmpliSens®, Russia) and stored at −70°C until evaluation.

### 2.2 Molecular studies based on real-time PCR

Nucleic acids from tick samples were analyzed for the presence of TBEV using the RealBest® RNA TBEV real-time PCR kit (Vector-Best, Novosibirsk, Russia) in accordance with manufacturer instructions. The study was conducted using the CFX96 C1000 TouchTM (Bio-Rad, USA) instrument.

### 2.3 Library preparation and sequencing of tick-borne encephalitis virus

Ten samples were genotyped on the Illumina MiSeq high-throughput sequencing platform (Illumina, California, USA) using MiSeq Reagent Kit v.3 (Illumina, California, USA). Library preparation was carried out in accordance with the protocol of the KAPA RNA HyperPrep Kit (Roche, USA) following manufacturer recommendations. Total RNA concentration was measured with a Qubit 4.0 Fluorimeter (Invitrogen, Waltham, MA, USA) and the Qubit RNA HS Assay Kit (Invitrogen, Waltham, MA, USA). Ten samples met the recommendations for reliable further analysis with total RNA concentrations ranging from 10 to 15 ng.

### 2.4 Phylogenetic analysis

In addition to the TBEV isolates sequenced, certain additional GenBank database sequences were analyzed. These were TBEV E protein sequences 1105 bp in length (genomic region 1173–2277). A total of 106 additional sequences were identified, including those for TBEV-Eu, TBEV-FE, and TBEV-Sib. To construct a phylogenetic tree, sequence alignment was performed using the ClustalW method with MEGA 11 software [13]. The general time reversible model (GTR), which was determined using the “Find best model DNA” algorithm, was selected as the optimal model. The phylogenetic tree was constructed using MEGA 11. The reliability of branching was assessed using bootstrap analysis (1000 bootstrap replicas). A pairwise distance matrix was calculated for E protein gene sequences, as the proportion of differing sites from a set of 11 samples, using the ape R package [14]. The dendrogram was constructed by applying hierarchical clustering using hclust with pairwise distances and sorted using the dendsort R package [15, 16]. The pheatmap R package was used for pheatmap visualization.

### 2.5 Population analysis

In order to perform population analysis, we applied the clusteron approach (CA) [17] provided by TBEV Analyzer 3.0 (available at tbev.viroinformatics.com) [18, 19]. The CA algorithm relies on a specific 454 bp glycoprotein E fragment (from nt 309 to 762 according to the sequence of the Vasilchenko strain, GenBank: M97369) [20]. It infers the phylogenetic characteristics of a query sequence from a network at both global and local scales, generating a three-fold output including subtype, phylogenetic lineage, and clusteron. The smallest population unit in CA is called a clusteron (derived from “cluster” and “clone”). It is typically defined as a group of strains with an identical amino acid sequence (signature) of the E protein fragment, which is phylogenetically related and characterized by a specific type of spatial distribution.

## 3. Results

### 3.1 TBEV prevalence in ticks

Analysis of tick species composition revealed a predominance of *I*. *persulcatus* in the Arkhangelsk region, the Leningrad region, and the Republic of Karelia. In the current study, the Arkhangelsk region and the Republic of Karelia showed an absolute dominance of *I*. *persulcatus*. In the Leningrad region, 16% of all studied ticks belonged to *I*. *ricinus*. In the Pskov region, the Komi Republic, and Saint Petersburg, a predominance of *I*. *ricinus* was revealed: 78% in the Pskov region; 63% in the Komi Republic; and 52% in Saint Petersburg.

Analysis of collected ticks by developmental stage revealed an absolute predominance of adult ticks (n = 2744, 98%). Sixty-eight pools of nymphs (2%) were collected and studied (from 2 to 25 individuals in each pool), without any detection of TBEV. However, the average TBEV prevalence was high (S2 Table), detected in 68 (2.4%) adults, including 61 *I*. *persulcatus* (3.2%) and 7 *I*. *ricinus* (0.8%). We detected TBEV RNA in ticks collected at 20 out of 93 sampling sites (22%) located in northwestern Russia.

The highest TBEV prevalence (6.1%) was detected in the Republic of Karelia. Ticks infected with TBEV were found in seven out of eight surveyed sampling sites (sampling numbers 70–73, 75–77, S1 Table). In the Arkhangelsk region, the prevalence was slightly lower (5.4%), detecting infected ticks with TBEV in 5 out of 11 surveyed sites (No. 1, 3, 4, 6, 11). In the Sysolsky district (Komi Republic), TBEV prevalence was 2% (site No. 81). In the Leningrad region, infected ticks were found in 2 out of 27 surveyed sites (No. 14, 22), with an average TBEV prevalence of around 1.4%. In Saint Petersburg, ticks infected with TBEV were found in 4 out of 10 surveyed spots (No. 85, 87, 88, 90), with a prevalence of around 0.5%. In the Pskov region, infected ticks with a prevalence of 0.4% were detected only in the Ostrovsky district (site No. 60).

### 3.2 TBEV genotyping

**Fig 2:**
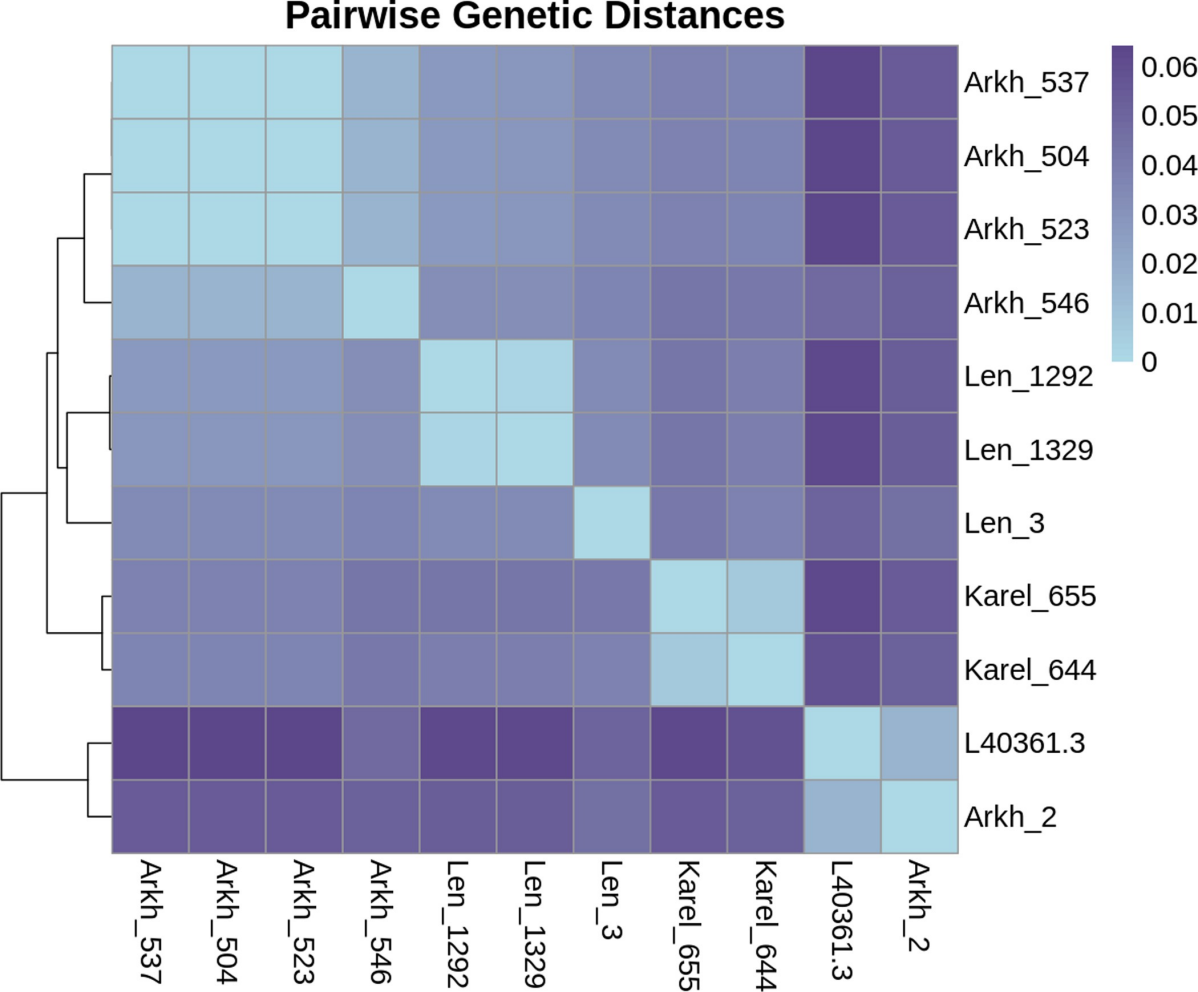
Pairwise genetic distance heatmap of ten E protein sequences. Each cell shows the genetic distance between pairs of sequences (versus x-axis). The genetic distances are color-coded, ranging from dark purple (indicating greater divergence) to light blue (indicating high similarity). The arrangement of samples on the heatmap is determined by hierarchical clustering.

To assess the genetic diversity of TBEV in northwestern Russia, structural protein genes (E protein) of 10 TBEV strains isolated in the Leningrad region (three samples), in the Republic of Karelia (two samples), and in the Arkhangelsk region (five samples) were analyzed. The heatmap visually represents the pairwise genetic distances of the reference genome (L40361) and ten E protein gene sequences from northwest Russia (Fig 2). As expected, genetic distances between the sequences of TBEV isolates from the Leningrad region, the Republic of Karelia, and the Arkhangelsk region allow their classification into distinct clusters. There are close relative genetic distances between all the isolated sequences obtained in the study (<0.06). However, they are distant from the reference sequence (>0.05), except Arkh_2. These two strains (L40361.3, Arkh_2) exhibit a close genetic relationship, forming a distinct branch in the hierarchical clustering. The second branch consists of three subgroups (Arkh, Len, Karel) which form a group of related clusters on the heatmap. Arkh_2 does not group with the Arkh cluster, suggesting that it has derived from a separate lineage and is more closely related to L40361.3. In the Arkh subgroup (Arkh_504, Arkh_523, Arkh_537, Arkh_546), genetic distances vary from 0.00 to 0.02. The Len subgroup (Len_3, Len_1292, Len_1329) shows distances between 0.00 and 0.03, while the Karel subgroup samples (Karel_655, Karel_644) are distinct by 0.01.

**Fig 3:**
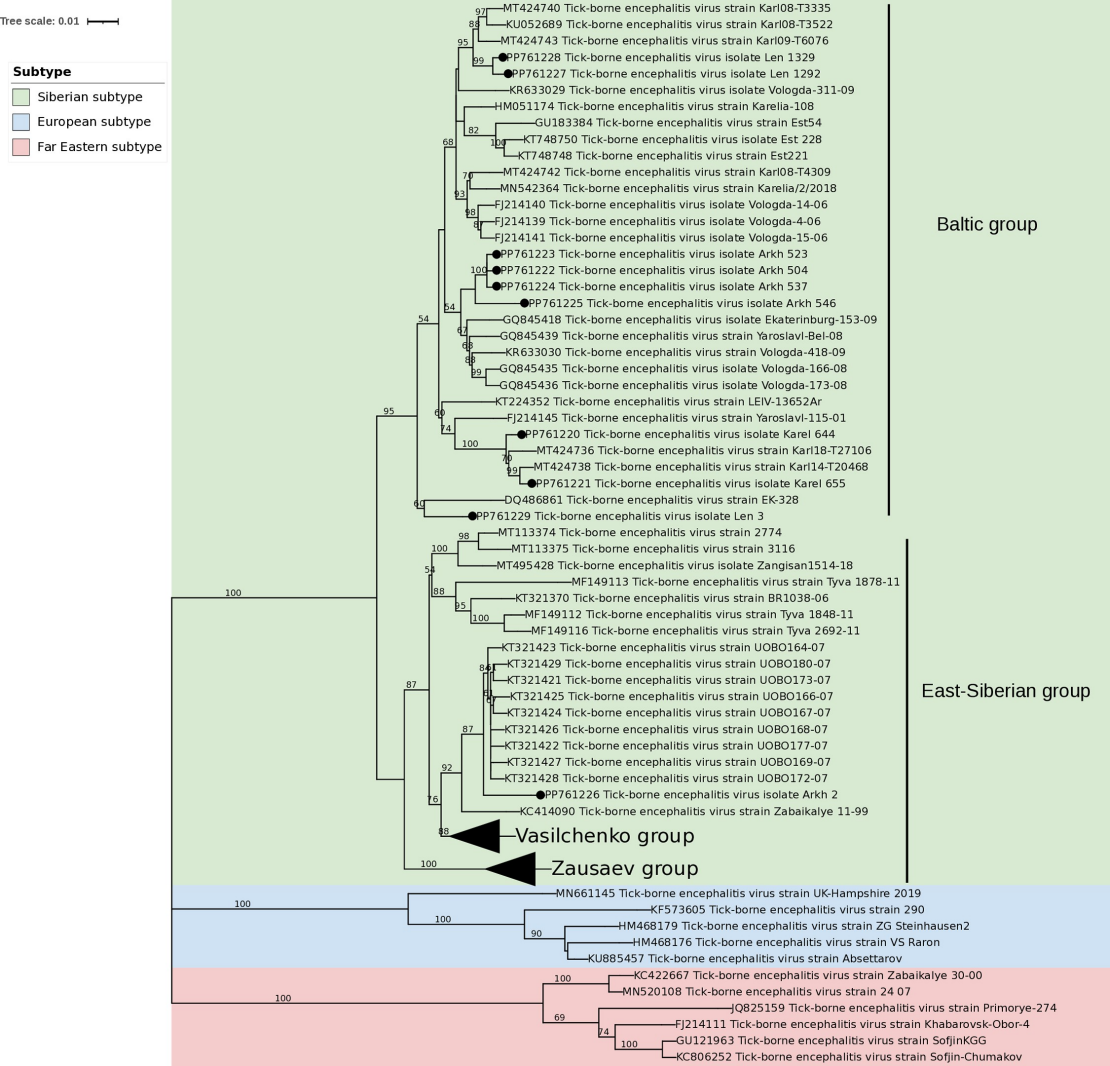
Phylogenetic tree of tick-borne encephalitis virus E protein. The percentage of trees in which the associated taxa clustered together is shown next to the branches. The tree is drawn to scale, with branch lengths measured in the number of substitutions per site (next to the branches). This analysis involved 116 nucleotide sequences. There were a total of 1105 bp in the final dataset. Sequences obtained in this study are highlighted with a black dot.

The phylogenetic tree of TBEV can be grouped into three distinct clusters corresponding to European, Siberian, and Far Eastern subtypes. The differences between these subtypes are clear. All sequenced TBEV isolates belong to the Siberian subtype, divided into the Baltic and Vasilchenko groups (Fig 3). The Baltic group included clusters of TBEV isolates from the Republic of Karelia (Karel_644, Karel_655), the Arkhangelsk region (Arkh_504, Arkh_523, Arkh_537, Arkh_546), and the Leningrad region (Len_1292, Len_1329). The Vasilchenko group included one isolate from the Arkhangelsk region (Arkh_2).

TBEV isolates Karel_644 and Karel_655 form a cluster which grouped with previous isolates from the Republic of Karelia (MT424736, MT424738). Isolates Arkh_504, Arkh_523, Arkh_537, and Arkh_546 were clustered with isolates obtained earlier in the Vologda (KR633030, GQ845435, GQ845436), Yaroslavl (GQ845439), and Sverdlovsk (GQ845418) regions. TBEV isolates Len_1292 and Len_1329 are grouped with isolates from the Republic of Karelia (MT424740, KU052689, MT424743). A separate branch was formed by a strain from the Leningrad region (Len_3) (Fig 3). TBEV isolate Arkh_2, which is part of the Vasilchenko group, clusters with isolates obtained earlier in the Irkutsk region (KT321421—KT321429).

**Fig 4:**
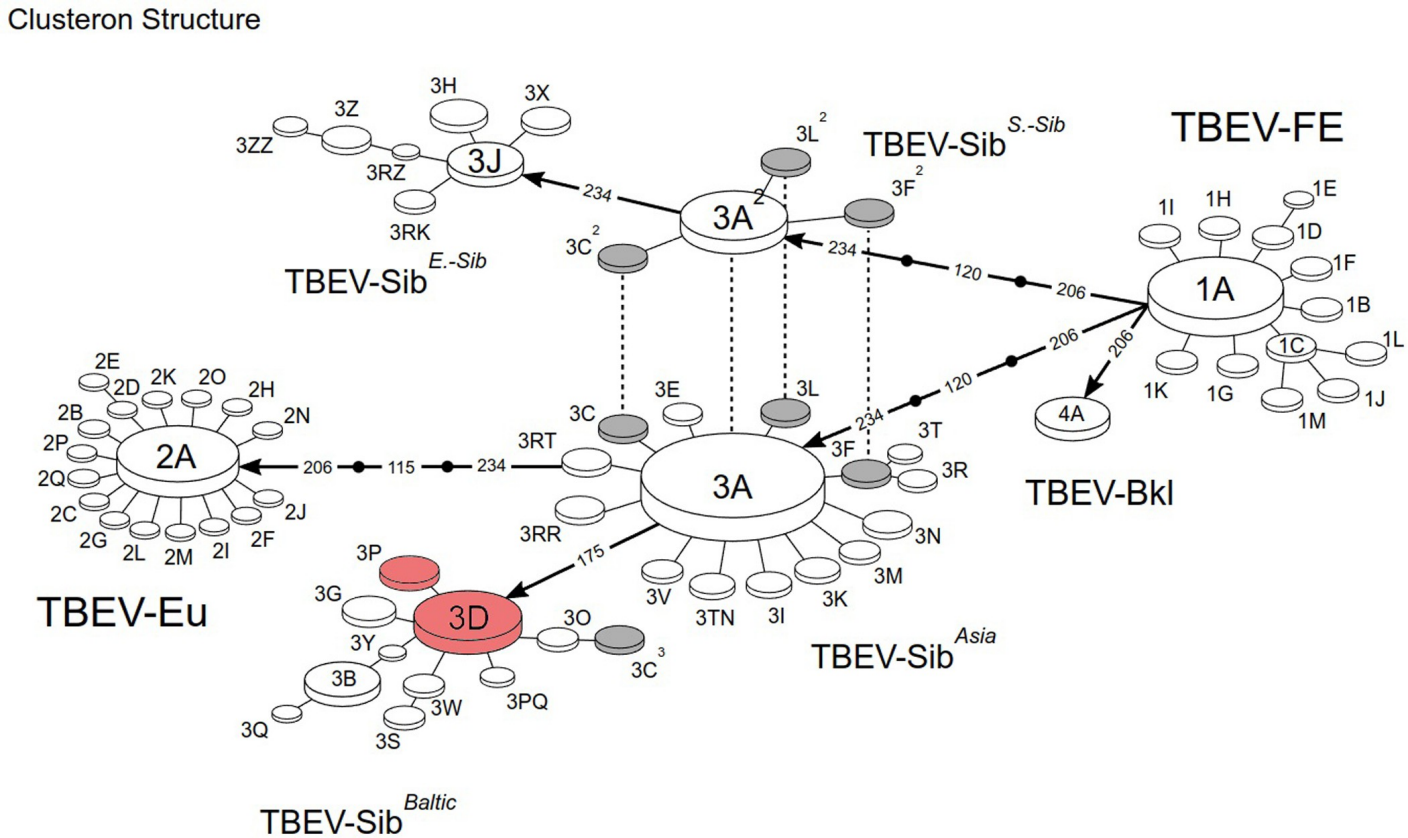
TBEV clusteron structure. The figure shows the distribution of clusters within TBEV genetic variants. The clusters that included the studied samples are marked in red.

**Fig 5:**
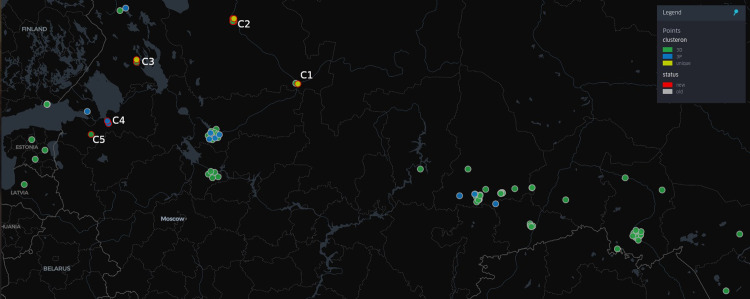
Distribution of viruses associated with clusterons 3D and 3P. Founder clusteron 3D strains are marked in green, whereas those for clusteron 3P are in blue. Our unique viruses are highlighted in yellow. The coordinates customized with the red outline are the location of our samples, whereas those with a white outline are obtained from the map of TBEV analyzer. Location C1 is related to Arkh_2, whereas C2 includes the rest of the samples from the Arkhangelsk region (Arkh_537, Arkh_504, Arkh_523, Arkh_546). Coordinates in C3 illustrate samples from the Republic of Karelia (Karel_655, Karel_644). Location C4 is associated with Len_1292 and Len_1329, whereas the final location C5 represents Len_3.

Table 1 shows the phylogenetic characteristics of query strains as well as their geographical coordinates. The majority of the strains cluster with the Siberian subtype. The Baltic phylogenetic lineage, 3D, seems to be dominant and is observed in all three regions (Arkhangelsk, Republic of Karelia, Leningrad). Five of the ten strains belong to this clusteron. This is not unexpected since clusteron 3D is the founder of the Baltic lineage and has a widespread distribution (Fig 4). Two strains from the Leningrad region were determined to belong to clusteron 3P, which is a first-degree clusteron derived from its founder, 3D. Fig 5 illustrates the distribution of clusterons 3D and 3P along with our samples.

**Table 1:** Characteristics of ten TBEV isolates.

Name	Subtype	Lineage	Clusteron	Coordinates
Arkh_537	Siberian	Baltic	3D	63°37’28.0"N 41°37’32.0"E
Arkh_504	Siberian	Baltic	3D	63°37’28.0"N 41°37’32.0"E
Arkh_523	Siberian	Baltic	3D	63°37’28.0"N 41°37’32.0"E
Len_3	Siberian	Baltic	3D	59°28’41.0"N 30°17’29.0"E
Karel_644	Siberian	Baltic	3D	62°03’17.0"N 33°57’42.0"E
Len_1292	Siberian	Baltic	3P	59°48’42.0"N 31°39’41.0"E
Len_1329	Siberian	Baltic	3P	59°48’42.0"N 31°39’41.0"E
Arkh_2	Siberian	East-Siberian	unique	61°15’00.0"N 46°39’00.0"E
Arkh_546	Siberian	Baltic	unique	63°37’28.0"N 41°37’32.0"E
Karel_655	Siberian	Baltic	unique	62°03’17.0"N 33°57’42.0"E

Phylogenetic analysis was performed using TBEV Analyzer 3.0. All viruses belong to the Siberian subtype. The majority of viruses belong to the Baltic lineage and clusteron 3D.

TBEV Analyzer determined the last three strains (Arkh_2, Arkh_546, Karel_655) from Table 1 to be unique. Arkh_2 belongs to the East-Siberian lineage, whereas Karel_655 and Arkh_546 are associated with the Baltic lineage. The study identified three isolates that are unique, which might represent new clusters.

## 4. Discussion

Two TBEV tick vectors, *I*. *ricinus* and *I*. *persulcatus*, have a habitat in northwestern Russia. The current results indicate an absolute dominance of *I*. *persulcatus* in the Arkhangelsk region and the Republic of Karelia, which is in line with previously published data [21–23]. In the European region of Russia, there has been a migration of ixodid ticks to the North in recent decades, largely due to climate change [24]. The obtained results on the prevalence of *I*. *ricinus* ticks St. Petersburg and the Komi Republic differ from previously obtained data [23, 25]. This may be due to differences in tick collection sites.

The average prevalence of questing ticks with TBEV in the studied regions exceeds the previous average for Russia by 3-fold: 2.4% versus 0.78% [10]. However, this number is close to the prevalence for the Baltic countries [21–26]. In the northwestern regions, the prevalence of *I*. *persulcatus* with TBEV was higher than that of *I*. *ricinus* (3.2% and 0.8%, respectively), which is consistent with the data of other authors [27]. The high prevalence of TBEV in ticks obtained in the Republic of Karelia and the Arkhangelsk region correlates with the high TBE incidence in these regions [10].

According to the results of this study, the number of infected ticks in St. Petersburg is slightly higher than found by other authors (0.5% versus 0.14% and 0.3%) [23, 28]. However, the number of ticks infected with TBEV in the Arkhangelsk Region and the Komi Republic is lower than previously published data (5.4% versus 7.8%; and 2% versus 5.2 and 6.8%, respectively) [21, 24, 29]. The lower level of TBEV infection in local ticks in Arkhangelsk Oblast and the Komi Republic may be related to the smaller number of administrative districts surveyed in this study. It is possible that a more widespread sampling would have yielded results more in line with previous findings. In addition, the geographical location of tick collection points, and their number, differed in each administrative district. Similarly, the present number of infected ticks in the Republic of Karelia and the Pskov region generally corresponds to previously obtained results [23, 30].

In our opinion, data on the localization of ticks infected with TBEV make it possible to increase the effectiveness of preventive measures. The detection of TBEV in the northern latitudes of the Republic of Karelia argues for the possibility of preventive measures in this region, especially since local residents are more vulnerable to TBEV if they have not previously been infected [31].

The genetic diversity data obtained indicate that the Siberian subtype, TBEV-Sib, is dominant in northwestern Russia. This is consistent with previously published data on TBEV genotyping in these regions, including border areas [32–34]. TBEV-Sib, containing at least five different lineages (Zausaev, Vasilchenko, Baltic, Obskaya, Bosnian), is the most genetically heterogeneous among the known subtypes [35]. Each lineage has a specific geographical distribution [35–40]. Detection of only Siberian subtype virus in northwestern Russia suggests extensive expansion of this subtype westward and probable displacement of European genotype strains. This is likely due to the fact that the spread of TBEV-Sib in natural conditions is carried out by two species of ticks, *I*. *persulcatus* and *I* .*ricinus*, which distinguishes this subtype from the European and Far Eastern ones. Features include a pronounced viral persistence, which is most often observed in infected animals on which the ticks feed. There is a possible connection with changes in climatic factors, as well as with anthropogenic impacts on the environment, in particular, with the transformation of natural landscapes. The Siberian subtype is associated with more severe disease (fatality rate of 1–3%), with a tendency for patients to develop chronic or extremely prolonged infections [41].

Phylogenetic analysis showed that the samples analyzed in this study belong to two groups: Baltic and Vasilchenko TBEV-Sib. Within the Baltic group, there was a division into different clusters. Strains from the Republic of Karelia and the Leningrad region formed common clusters. This may be due to the presence of persistent perennial foci in the Republic of Karelia [34]. This is confirmed by the minimum genetic distances between strains. In addition, isolates from the Arkhangelsk region clustered with isolates from the Vologda, Yaroslavl, and Sverdlovsk regions. This confirms the previously described absence of a direct relationship between genetic and geographic distance in different strains of the TBEV Baltic group [38, 42].

Analysis of the obtained data showed that genetically similar variants of the virus could be observed in remote areas. In our study, the Arkh_2 isolate from the Arkhangelsk region belongs to the Vasilchenko group, which is more typical for the Irkutsk region [43]. This may be due to the participation of migratory birds in the spread of TBEV over long distances [7].

It is known that all clusterons of the Baltic lineage have asparagine (N) at position 175 in their clusteron-specific amino acid signature, with the exception of clusteron 3C^3^, which is homoplastic, as defined earlier [40]. The only difference between the signatures of clusterons 3D and 3P occurs at position 234, where histidine (H) in 3D is replaced by tyrosine (Y) in 3P [20]. The results of phylogenetic analysis based on amino acid structure using the clusteron framework indicate that the majority of isolates belong to clusteron 3D (founder of the Baltic phylogenetic lineage). Use of the CA can help in assessing the variability of the virus within a genetic lineage at the amino acid level.

Three sequences were unique, yet bearing similarities to GenBank submissions, suggesting the formation of new candidate clusterons. Unique strains may play an important role in TBEV surveillance, as they may help identify the formation of a new outbreak or genetic variant of the virus. In addition to having unique phylogenetic characteristics and a specific amino acid signature, declaring a new clusteron in the CA requires enough evidence about its stability. One such requirement is the existence of at least two or three viruses with the same characteristics. In the final step, a new candidate clusteron is manually verified by the TBEV Analyzer team, characterized, named, registered, and added to the clusteron structure [19]. It is worth mentioning that adding a new clusteron to the phylogenetic structure mainly depends on a comprehensive analysis of the phylogenetic network, which is out of the scope of this work.

Identification and characterization of genetic lineages within TBEV subtypes provide insight into changes associated with viral evolution in endemic areas. Data on lineages and their properties may aid in future studies with comprehensive assessments of tick-borne encephalitis foci. The discovery of strains representing potential founders of new clusterons argues in favor of ongoing viral evolution.

## 5. Conclusion

*Ixodes persulcatus* is the primary vector of TBEV in northwest Russia. The high level of TBEV infection in local ticks detected in the Arkhangelsk and Karelia regions correlates with the high registered incidence of TBE in those areas. Currently, we are witnessing the westward expansion of the Siberian TBEV subtype, with distribution to new regions in Eastern and Western Europe. The genetic diversity of new clusters confirms the ongoing active evolution of TBEV. Further studies using whole-genome sequencing are necessary for a better understanding of the regional genetic diversity of TBEV strains.

## Supporting information

S1 Table Number and collection points of ticks, 2015–2022. (DOCX)

S2 Table Results of tick testing for TBEV infection. (DOCX)
