# Supplementary material for: Prevalence and genetic diversity of tick-borne encephalitis virus in ixodid ticks from specific regions of northwestern Russia
Source: PLoS One. 2025 Jan 30;20(1):e0314385. doi: 10.1371/journal.pone.0314385 (PMC11781730; doi:10.1371/journal.pone.0314385)
Supplement: S1 Table — (DOCX) [file pone.0314385.s001.docx]

| **Subject of the Russian Federation** | **Administrative region** | **Locality** | **Number of the tick collection site** | **Coordinates** | **Total collected** | **including** | |
| --- | --- | --- | --- | --- | --- | --- | --- |
|  |  |  |  |  |  | ***I. ricinus*** | ***I. persulcatus*** |
| **Arkhangelsk region** | Velsky | vil. Alexandrovskaya | 1 | 61°07'33.0"N 41°59'05.0"E | 79 | 0 | 79 |
|  |  | vil. Solginsky | 2 | 61°02'59.0"N 41°20'35.0"E | 5 | 0 | 5 |
|  | Verkhnetoyemsky | vil. Dvinskaya | 3 | 62°09'17.0"N 45°07'11.0"E | 7 | 0 | 7 |
|  | Vinogradovsky | vil. Bereznik | 4 | 62°51'00.0"N 42°45'00.0"E | 95 | 0 | 95 |
|  | Konoshsky | vil. Ivakinskaya | 5 | 61°03'41.2"N 40°30'43.3"E | 7 | 0 | 7 |
|  | Kotlassky | Kotlas | 6 | 61°15'00.0"N 46°39'00.0"E | 109 | 0 | 109 |
|  | Krasnoborsky | vil. Krasnoborsk | 7 | 61°33'00.0"N 45°56'00.0"E | 6 | 0 | 6 |
|  | Ustyansky | vil. Malinovka | 8 | 61°07'39.6"N 43°20'54.8"E | 27 | 0 | 27 |
|  | Kholmogorsky | vil. Bryn-Navolok | 9 | 63°44'05.0"N 41°24'34.0"E | 1 | 0 | 1 |
|  |  | vil. Palovo | 10 | 63°52'24.5"N 41°29'13.7"E | 2 | 0 | 2 |
|  |  | vil. Usolye | 11 | 63°37'28.0"N 41°37'32.0"E | 105 | 0 | 105 |
| **Total:** | | | | | **443** | **0** | **443** |
| **Leningrad region** | Boksitogorsky | vil. Astrachi | 12 | 59°37'26.0"N 33°39'28.0"E | 9 | 0 | 9 |
|  | Vsevolozhskiy | vil. Morozova | 13 | 59°58'28.0"N 31°02'06.0"E | 3 | 0 | 3 |
|  |  | vil. Chernaya Rechka | 14 | 59°55'17.0"N 30°58'45.0"E | 18 | 0 | 18 |
|  |  | vil. Yubileiny-Ruchi | 15 | 60°13'03.0"N 30°31'22.1"E | 30 | 30 | 0 |
|  | Gatchinsky | vil. Virkino | 16 | 59°28'41.0"N 30°17'29.0"E | 11 | 0 | 11 |
|  |  | vil. Krasnitsy | 17 | 59°27'08.0"N 30°20'56.0"E | 6 | 1 | 5 |
|  |  | vil. Pustoshka | 18 | 59°30'45.0"N 30°12'43.0"E | 1 | 0 | 1 |
|  | Kingiseppsky | vil. Kaskolovka | 19 | 59°23'20.1"N 28°38'34.0"E | 8 | 0 | 8 |
|  |  | vil. Kotly | 20 | 59°36'21.0"N 28°45'18.0"E | 43 | 4 | 39 |
|  |  | Kingisepp | 21 | 59°22'41.0"N 28°36'04.4"E | 6 | 0 | 6 |
|  | Kirovsky | Lava River | 22 | 59°48'42.0"N 31°39'41.0"E | 105 | 2 | 103 |
|  | Lomonosovsky | vil. Kipen | 23 | 59°41'05.0"N 29°51'04.0"E | 21 | 21 | 0 |
|  |  | place Nizhnee Koirovo | 24 | 59°47'15.8"N 30°16'16.7"E | 3 | 0 | 3 |
|  | Luzhsky | vil. Kamenka | 25 | 58°49'09.0"N 30°05'50.0"E | 1 | 0 | 1 |
|  | Podporozhsky | vil. Vinnytsia | 26 | 60°37'42.0"N 34°46'25.0"E | 10 | 0 | 10 |
|  |  | vil. Kiprushino | 27 | 60°56'35.0"N 35°25'48.0"E | 8 | 0 | 8 |
|  |  | Podporozhye | 28 | 60°54'44.0"N 34°10'05.0"E | 1 | 0 | 1 |
|  | Priozersky | vil. Michurinskoe | 29 | 60°33'54.0"N 29°52'19.0"E | 2 | 1 | 1 |
|  | Slantsevsky | vil. Vyskatka | 30 | 59°01'14.0"N 28°11'01.0"E | 13 | 0 | 13 |
|  |  | vil. Medvezhek | 31 | 59°11'19.0"N 28°20'58.0"E | 24 | 0 | 24 |
|  | Tikhvinsky | vil. Kayvaksa | 32 | 59°43'09.0"N 33°26'29.0"E | 1 | 0 | 1 |
|  |  | vil. Melegezhskaya Gorka | 33 | 59°34'02.0"N 33°24'57.0"E | 9 | 0 | 9 |
|  |  | vil. Ust-Kapsha | 34 | 59°52'37.0"N 33°43'26.0"E | 13 | 0 | 13 |
|  | Tosnensky | vil. Eglizi | 35 | 59°32'41.0"N 30°42'14.0"E | 12 | 0 | 12 |
|  |  | vil. Novolisino | 36 | 59°33'50.0"N 30°46'01.0"E | 3 | 0 | 3 |
|  |  | Lisinskoe rural settlement | 37 | 59°29'02.9"N 30°34'57.0"E | 5 | 0 | 5 |
|  |  | Urban-type settlement Fornosovo | 38 | 59°34'09.0"N 30°33'04.0"E | 1 | 0 | 1 |
| **Total:** | | | | | **367** | **59** | **308** |
| **Pskov region** | Velikoluksky | Velikie Luki | 39 | 56°21'00.0"N 30°31'00.0"E | 1 | 1 | 0 |
|  |  | Dacha array | 40 | 56°21'00.0"N 30°31'00.0"E | 1 | 0 | 1 |
|  |  | lake Kisloye, recreational area | 41 | 56°22'35.0"N 30°21'59.0"E | 1 | 0 | 1 |
|  |  | lake Hamshonets, recreational area | 42 | 56°22'45.5"N 30°23'21.3"E | 2 | 1 | 1 |
|  |  | vil. Pereslegino | 43 | 56°23'03.0"N 30°26'17.0"E | 1 | 0 | 1 |
|  |  | lake Porechenskoe, recreational area | 44 | 56°06'40.0"N 30°29'21.0"E | 1 | 1 | 0 |
|  |  | Senchita forest | 45 | 56°10'21.9"N 30°38'54.0"E | 3 | 0 | 3 |
|  | Dnovsky | vil. Vyskod | 46 | 57°46'24.0"N 30°04'33.0"E | 15 | 15 | 0 |
|  |  | Dno | 47 | 57°50'00.0"N 29°58'00.0"E | 3 | 3 | 0 |
|  | Krasnogorodskiy | Urban-type settlement Krasnogorodsk | 48 | 56°49'44.0"N 28°16'51.0"E | 5 | 5 | 0 |
|  | Kuninskiy | vil. Zhizhitsa | 49 | 56°16'47.0"N 31°21'14.0"E | 1 | 0 | 1 |
|  |  | lake Zizhitskoe, recreational area | 50 | 56°13'53.0"N 31°15'05.0"E | 1 | 1 | 0 |
|  |  | vil. Naumovo | 51 | 56°16'33.0"N 31°19'24.0"E | 2 | 0 | 2 |
|  | Loknyanskiy | vil. Valuevskoye | 52 | 56°44'36.0"N 30°10'53.0"E | 2 | 0 | 2 |
|  |  | vil. Sukhlovo | 53 | 56°40'18.0"N 30°08'01.0"E | 2 | 0 | 2 |
|  | Nevelsky | Nevel | 54 | 56°01'00.0"N 29°56'00.0"E | 2 | 2 | 0 |
|  | Novorzhevskiy | vil. Zhadritsy | 55 | 56°55'43.0"N 29°09'38.0"E | 7 | 7 | 0 |
|  | Novosokolnichesky | vil. Nasva | 56 | 56°34'35.0"N 30°08'45.0"E | 1 | 0 | 1 |
|  |  | vil. Olokhovo | 57 | 56°21'01.0"N 30°17'03.0"E | 2 | 0 | 2 |
|  |  | lake Sapozhek, recreational area | 58 | 56°20'58.5"N 30°21'00.4"E | 1 | 1 | 0 |
|  | Opochetskiy | Opochka | 59 | 56°43'00.0"N 28°39'00.0"E | 9 | 9 | 0 |
|  | Ostrovsky | lake Gorokhovo, forest area | 60 | 57°16'34.0"N 28°21'19.0"E | 8 | 6 | 2 |
|  | Plusskiy | Urban-type settlement Plussa | 61 | 58°25'44.0"N 29°21'36.0"E | 1 | 0 | 1 |
|  | Pskovsky | Pskov | 62 | 57°49'00.0"N 28°20'00.0"E | 2 | 0 | 2 |
|  | Pustoshkinsky | vil. Avinishchi | 63 | 56°04'42.0"N 29°15'59.0"E | 3 | 0 | 3 |
|  |  | Pustoshka | 64 | 56°20'00.0"N 29°22'00.0"E | 3 | 0 | 3 |
|  |  | vil. Kholyuny | 65 | 56°25'39.0"N 29°09'34.0"E | 7 | 7 | 0 |
|  | Pushkinogorskiy |  | 66 | 57°01'00.0"N 28°55'00.0"E | 42 | 20 | 22 |
|  | Sebezhsky | vil. Ilovo | 67 | 56°16'22.0"N 28°27'00.0"E | 48 | 48 | 0 |
|  |  | Recreation center Ozeryavki | 68 | 56°11'42.4"N 28°29'04.1"E | 34 | 34 | 0 |
|  | Strugokrasnenskiy | Urban-type settlement Strugi Krasnyye | 69 | 58°16'20.0"N 29°06'30.0"E | 12 | 12 | 0 |
| **Total:** | | | | | **223** | **173** | **50** |
| **Republic of Karelia** | Kondopozhsky | vil. Gomselga | 70 | 62°03'17.0"N 33°57'42.0"E | 125 | 0 | 125 |
|  | Medvezhyegorsky |  | 71 | 62°55'00.0"N 34°28'00.0"E | 63 | 0 | 63 |
|  | Petrozavodsk city district | Petrozavodsk | 72 | 61°47'46.0"N 34°20'57.0"E | 29 | 0 | 29 |
|  |  | Petrozavodsk, Botanical Garden | 73 | 61°50'57.0"N 34°19'54.0"E | 37 | 0 | 37 |
|  | Prionezhskiy | vil. Verkhovye | 74 | 61°55'26.0"N 34°12'07.0"E | 28 | 0 | 28 |
|  |  | place Chortov stul | 75 | 54°49'45.3"N 41°11'56.2"E | 37 | 0 | 37 |
|  | Pryazhinsky | vil. Mishinselga | 76 | 61°43'19.0"N 33°09'10.0"E | 138 | 0 | 138 |
|  | Segezhsky |  | 77 | 63°44'00.0"N 34°19'00.0"E | 21 | 0 | 21 |
| **Total:** | | | | | **478** | **0** | **478** |
| **Komi Republic** | Koygorodskiy |  | 78 | 60°27'33.0"N 51°00'37.0"E | 1 | 0 | 1 |
|  | Priluzskiy |  | 79 | 60°20'19.0"N 49°36'33.0"E | 2 | 0 | 2 |
|  | Syktyvdinsky |  | 80 | 61°37'21.0"N 50°45'26.0"E | 29 | 19 | 10 |
|  | Sysolsky |  | 81 | 61°05'00.0"N 50°05'00.0"E | 167 | 108 | 59 |
|  | Ust-Vymskiy |  | 82 | 62°13'31.0"N 49°59'40.0"E | 1 | 0 | 1 |
|  | Ust-Kulomskiy |  | 83 | 61°41'00.0"N 53°40'00.0"E | 2 | 0 | 2 |
| **Total:** | | | | | **202** | **127** | **75** |
| **Saint Petersburg** | Kurortniy | Cottage village Zhemchuzhina Razliva | 84 | 60°03'38.3"N 29°59'57.7"E | 219 | 3 | 216 |
|  |  | vil. Molodezhnoe | 85 | 60°11'40.0"N 29°31'30.0"E | 5 | 5 | 0 |
|  |  | vil. Repino | 86 | 60°10'08.0"N 29°52'20.0"E | 3 | 0 | 3 |
|  |  | vil. Serovo | 87 | 60°12'16.0"N 29°33'48.0"E | 169 | 166 | 3 |
|  |  | vil. Smolyachkovo | 88 | 60°10'48.0"N 29°28'33.0"E | 162 | 162 | 0 |
|  |  | vil. Smolyachkovo | 89 | 60°10'48.0"N 29°28'33.0"E | 94 | 90 | 4 |
|  |  | vil. Smolyachkovo | 90 | 60°10'48.0"N 29°28'33.0"E | 132 | 131 | 1 |
|  |  | vil. Solnechnoe | 91 | 60°08'56.0"N 29°56'12.0"E | 18 | 0 | 18 |
|  | Primorskiy | vil. Lisiy Nos | 92 | 60°00'54.0"N 30°00'30.0"E | 294 | 17 | 277 |
|  |  | vil. Olgino | 93 | 60°00'02.8"N 30°08'23.3"E | 3 | 0 | 3 |
| **Total:** | | | | | **1099** | **574** | **525** |
| **Total:** | | | | | **2812** | **933** | **1879** |
