# Supplementary material for: Prevalence and genetic diversity of tick-borne encephalitis virus in ixodid ticks from specific regions of northwestern Russia
Source: PLoS One. 2025 Jan 30;20(1):e0314385. doi: 10.1371/journal.pone.0314385 (PMC11781730; doi:10.1371/journal.pone.0314385)
Supplement: S2 Table — (DOCX) [file pone.0314385.s002.docx]

| **Subject of the  Russian Federation** | **Administrative region** | **Number of the tick collection site** | **Total**  ***I. ricinus*** | **including "+"** | **%** | **Total  *I. persulcatus*** | **including "+"** | **%** | **Total ticks** | **Total “+”** | **%** |
| --- | --- | --- | --- | --- | --- | --- | --- | --- | --- | --- | --- |
|  |  |  |  |  |  |  |  |  |  |  |  |
| **Arkhangelsk region** | Velsky | 1 | 0 | 0 | 0 | 79 | 3 | 3,8 | 79 | 3 | 3,8 |
|  |  | 2 | 0 | 0 | 0 | 5 | 0 | 0,0 | 5 | 0 | 0,0 |
|  | Verkhnetoyemsky | 3 | 0 | 0 | 0 | 7 | 1 | 14,3 | 7 | 1 | 14,3 |
|  | Vinogradovsky | 4 | 0 | 0 | 0 | 95 | 12 | 12,6 | 95 | 12 | 12,6 |
|  | Konoshsky | 5 | 0 | 0 | 0 | 7 | 0 | 0,0 | 7 | 0 | 0,0 |
|  | Kotlassky | 6 | 0 | 0 | 0 | 109 | 4 | 3,7 | 109 | 4 | 3,7 |
|  | Krasnoborsky | 7 | 0 | 0 | 0 | 6 | 0 | 0,0 | 6 | 0 | 0,0 |
|  | Ustyansky | 8 | 0 | 0 | 0 | 27 | 0 | 0,0 | 27 | 0 | 0,0 |
|  | Kholmogorsky | 9 | 0 | 0 | 0 | 1 | 0 | 0,0 | 1 | 0 | 0,0 |
|  |  | 10 | 0 | 0 | 0 | 2 | 0 | 0,0 | 2 | 0 | 0,0 |
|  |  | 11 | 0 | 0 | 0 | 105 | 4 | 3,8 | 105 | 4 | 3,8 |
| **Total:** | | | **0** | **0** | **0** | **443** | **24** | **5,4** | **443** | **24** | **5,4** |
| **Leningrad region** | Boksitogorsky | 12 | 0 | 0 | 0 | 9 | 0 | 0 | 9 | 0 | 0,0 |
|  | Vsevolozhskiy | 13 | 0 | 0 | 0 | 3 | 0 | 0 | 3 | 0 | 0,0 |
|  |  | 14 | 0 | 0 | 0 | 18 | 1 | 5,6 | 18 | 1 | 5,6 |
|  |  | 15 | 30 | 0 | 0 | 0 | 0 | 0 | 30 | 0 | 0,0 |
|  | Gatchinsky | 16 | 0 | 0 | 0 | 11 | 0 | 0 | 11 | 0 | 0,0 |
|  |  | 17 | 1 | 0 | 0 | 5 | 0 | 0 | 6 | 0 | 0,0 |
|  |  | 18 | 0 | 0 | 0 | 1 | 0 | 0 | 1 | 0 | 0,0 |
|  | Kingiseppsky | 19 | 0 | 0 | 0 | 8 | 0 | 0 | 8 | 0 | 0,0 |
|  |  | 20 | 4 | 0 | 0 | 39 | 0 | 0 | 43 | 0 | 0,0 |
|  |  | 21 | 0 | 0 | 0 | 6 | 0 | 0 | 6 | 0 | 0,0 |
|  | Kirovsky | 22 | 2 | 0 | 0 | 103 | 4 | 3,9 | 105 | 4 | 3,8 |
|  | Lomonosovsky | 23 | 21 | 0 | 0 | 0 | 0 | 0 | 21 | 0 | 0,0 |
|  |  | 24 | 0 | 0 | 0 | 3 | 0 | 0 | 3 | 0 | 0,0 |
|  | Luzhsky | 25 | 0 | 0 | 0 | 1 | 0 | 0 | 1 | 0 | 0,0 |
|  | Podporozhsky | 26 | 0 | 0 | 0 | 10 | 0 | 0 | 10 | 0 | 0,0 |
|  |  | 27 | 0 | 0 | 0 | 8 | 0 | 0 | 8 | 0 | 0,0 |
|  |  | 28 | 0 | 0 | 0 | 1 | 0 | 0 | 1 | 0 | 0,0 |
|  | Priozersky | 29 | 1 | 0 | 0 | 1 | 0 | 0 | 2 | 0 | 0,0 |
|  | Slantsevsky | 30 | 0 | 0 | 0 | 13 | 0 | 0 | 13 | 0 | 0,0 |
|  |  | 31 | 0 | 0 | 0 | 24 | 0 | 0 | 24 | 0 | 0,0 |
|  | Tikhvinsky | 32 | 0 | 0 | 0 | 1 | 0 | 0 | 1 | 0 | 0,0 |
|  |  | 33 | 0 | 0 | 0 | 9 | 0 | 0 | 9 | 0 | 0,0 |
|  |  | 34 | 0 | 0 | 0 | 13 | 0 | 0 | 13 | 0 | 0,0 |
|  | Tosnensky | 35 | 0 | 0 | 0 | 12 | 0 | 0 | 12 | 0 | 0,0 |
|  |  | 36 | 0 | 0 | 0 | 3 | 0 | 0 | 3 | 0 | 0,0 |
|  |  | 37 | 0 | 0 | 0 | 5 | 0 | 0 | 5 | 0 | 0,0 |
|  |  | 38 | 0 | 0 | 0 | 1 | 0 | 0 | 1 | 0 | 0,0 |
| **Total:** | | | **59** | **0** | **0** | **308** | **5** | **1,6** | **367** | **5** | **1,4** |
| **Pskov region** | Velikoluksky | 39 | 1 | 0 | 0 | 0 | 0 | 0 | 1 | 0 | 0,0 |
|  |  | 40 | 0 | 0 | 0 | 1 | 0 | 0 | 1 | 0 | 0,0 |
|  |  | 41 | 0 | 0 | 0 | 1 | 0 | 0 | 1 | 0 | 0,0 |
|  |  | 42 | 1 | 0 | 0 | 1 | 0 | 0 | 2 | 0 | 0,0 |
|  |  | 43 | 0 | 0 | 0 | 1 | 0 | 0 | 1 | 0 | 0,0 |
|  |  | 44 | 1 | 0 | 0 | 0 | 0 | 0 | 1 | 0 | 0,0 |
|  |  | 45 | 0 | 0 | 0 | 3 | 0 | 0 | 3 | 0 | 0,0 |
|  | Dnovsky | 46 | 15 | 0 | 0 | 0 | 0 | 0 | 15 | 0 | 0,0 |
|  |  | 47 | 3 | 0 | 0 | 0 | 0 | 0 | 3 | 0 | 0,0 |
|  | Krasnogorodskiy | 48 | 5 | 0 | 0 | 0 | 0 | 0 | 5 | 0 | 0,0 |
|  | Kuninskiy | 49 | 0 | 0 | 0 | 1 | 0 | 0 | 1 | 0 | 0,0 |
|  |  | 50 | 1 | 0 | 0 | 0 | 0 | 0 | 1 | 0 | 0,0 |
|  |  | 51 | 0 | 0 | 0 | 2 | 0 | 0 | 2 | 0 | 0,0 |
|  | Loknyanskiy | 52 | 0 | 0 | 0 | 2 | 0 | 0 | 2 | 0 | 0,0 |
|  |  | 53 | 0 | 0 | 0 | 2 | 0 | 0 | 2 | 0 | 0,0 |
|  | Nevelsky | 54 | 2 | 0 | 0 | 0 | 0 | 0 | 2 | 0 | 0,0 |
|  | Novorzhevskiy | 55 | 7 | 0 | 0 | 0 | 0 | 0 | 7 | 0 | 0,0 |
|  | Novosokolnichesky | 56 | 0 | 0 | 0 | 1 | 0 | 0 | 1 | 0 | 0,0 |
|  |  | 57 | 0 | 0 | 0 | 2 | 0 | 0 | 2 | 0 | 0,0 |
|  |  | 58 | 1 | 0 | 0 | 0 | 0 | 0 | 1 | 0 | 0,0 |
|  | Opochetskiy | 59 | 9 | 0 | 0 | 0 | 0 | 0 | 9 | 0 | 0,0 |
|  | Ostrovsky | 60 | 6 | 0 | 0 | 2 | 1 | 50 | 8 | 1 | 12,5 |
|  | Plusskiy | 61 | 0 | 0 | 0 | 1 | 0 | 0 | 1 | 0 | 0,0 |
|  | Pskovsky | 62 | 0 | 0 | 0 | 2 | 0 | 0 | 2 | 0 | 0,0 |
|  | Pustoshkinsky | 63 | 0 | 0 | 0 | 3 | 0 | 0 | 3 | 0 | 0,0 |
|  |  | 64 | 0 | 0 | 0 | 3 | 0 | 0 | 3 | 0 | 0,0 |
|  |  | 65 | 7 | 0 | 0 | 0 | 0 | 0 | 7 | 0 | 0,0 |
|  | Pushkinogorskiy | 66 | 20 | 0 | 0 | 22 | 0 | 0 | 42 | 0 | 0,0 |
|  | Sebezhsky | 67 | 48 | 0 | 0 | 0 | 0 | 0 | 48 | 0 | 0,0 |
|  |  | 68 | 34 | 0 | 0 | 0 | 0 | 0 | 34 | 0 | 0,0 |
|  | Strugokrasnenskiy | 69 | 12 | 0 | 0 | 0 | 0 | 0 | 12 | 0 | 0,0 |
| **Total:** | | | **173** | **0** | **0** | **50** | **1** | **2** | **223** | **1** | **0,4** |
| **Republic of Karelia** | Kondopozhsky | 70 | 0 | 0 | 0 | 125 | 2 | 1,6 | 125 | 2 | 1,6 |
|  | Medvezhyegorsky | 71 | 0 | 0 | 0 | 63 | 7 | 11,1 | 63 | 7 | 11,1 |
|  | Petrozavodsk city district | 72 | 0 | 0 | 0 | 29 | 1 | 3,4 | 29 | 1 | 3,4 |
|  |  | 73 | 0 | 0 | 0 | 37 | 8 | 21,6 | 37 | 8 | 21,6 |
|  | Prionezhskiy | 74 | 0 | 0 | 0 | 28 | 0 | 0,0 | 28 | 0 | 0,0 |
|  |  | 75 | 0 | 0 | 0 | 37 | 1 | 2,7 | 37 | 1 | 2,7 |
|  | Pryazhinsky | 76 | 0 | 0 | 0 | 138 | 5 | 3,6 | 138 | 5 | 3,6 |
|  | Segezhsky | 77 | 0 | 0 | 0 | 21 | 5 | 23,8 | 21 | 5 | 23,8 |
| **Total:** | | | **0** | **0** | **0** | **478** | **29** | **6,1** | **478** | **29** | **6,1** |
| **Komi Republic** | Koygorodskiy | 78 | 0 | 0 | 0 | 1 | 0 | 0 | 1 | 0 | 0,0 |
|  | Priluzskiy | 79 | 0 | 0 | 0 | 2 | 0 | 0 | 2 | 0 | 0,0 |
|  | Syktyvdinsky | 80 | 19 | 0 | 0 | 10 | 0 | 0 | 29 | 0 | 0,0 |
|  | Sysolsky | 81 | 108 | 3 | 2,8 | 59 | 1 | 1,7 | 167 | 4 | 2,4 |
|  | Ust-Vymskiy | 82 | 0 | 0 | 0 | 1 | 0 | 0 | 1 | 0 | 0,0 |
|  | Ust-Kulomskiy | 83 | 0 | 0 | 0 | 2 | 0 | 0 | 2 | 0 | 0,0 |
| **Total:** | | | **127** | **3** | **2,4** | **75** | **1** | **1,3** | **202** | **4** | **2,0** |
| **Saint Petersburg** | Kurortniy | 84 | 3 | 0 | 0,0 | 216 | 0 | 0 | 219 | 0 | 0,0 |
|  |  | 85 | 5 | 0 | 0,0 | 0 | 0 | 0 | 5 | 1 | 20,0 |
|  |  | 86 | 0 | 0 | 0,0 | 3 | 0 | 0 | 3 | 0 | 0,0 |
|  |  | 87 | 166 | 1 | 0,6 | 3 | 0 | 0 | 169 | 1 | 0,6 |
|  |  | 88 | 162 | 2 | 1,2 | 0 | 0 | 0 | 162 | 2 | 1,2 |
|  |  | 89 | 90 | 0 | 0,0 | 4 | 0 | 0 | 94 | 0 | 0,0 |
|  |  | 90 | 131 | 1 | 0,8 | 1 | 0 | 0 | 132 | 1 | 0,8 |
|  |  | 91 | 0 | 0 | 0,0 | 18 | 1 | 5,6 | 18 | 0 | 0,0 |
|  | Primorskiy | 92 | 17 | 0 | 0,0 | 277 | 0 | 0 | 294 | 0 | 0,0 |
|  |  | 93 | 0 | 0 | 0,0 | 3 | 0 | 0 | 3 | 0 | 0,0 |
| **Total:** | | | **574** | **4** | **0,7** | **525** | **1** | **0,2** | **1099** | **5** | **0,5** |
| **Total:** | | | **933** | **7** | **0,8** | **1879** | **61** | **3,2** | **2812** | **68** | **2,4** |
